# RNA Modifications and RNA Metabolism in Neurological Disease Pathogenesis

**DOI:** 10.3390/ijms222111870

**Published:** 2021-11-01

**Authors:** Biswanath Chatterjee, Che-Kun James Shen, Pritha Majumder

**Affiliations:** 1 Institute of Molecular Biology, Academia Sinica, Taipei City 115, Taiwan; biswa@gate.sinica.edu.tw; 2 Institute of Molecular Medicine, College of Medicine, National Cheng Kung University, Tainan 704, Taiwan

**Keywords:** RNA modifications, RNA metabolism, brain development, neurodegenerative diseases, neurodevelopmental disorders

## Abstract

The intrinsic cellular heterogeneity and molecular complexity of the mammalian nervous system relies substantially on the dynamic nature and spatiotemporal patterning of gene expression. These features of gene expression are achieved in part through mechanisms involving various epigenetic processes such as DNA methylation, post-translational histone modifications, and non-coding RNA activity, amongst others. In concert, another regulatory layer by which RNA bases and sugar residues are chemically modified enhances neuronal transcriptome complexity. Similar RNA modifications in other systems collectively constitute the cellular epitranscriptome that integrates and impacts various physiological processes. The epitranscriptome is dynamic and is reshaped constantly to regulate vital processes such as development, differentiation and stress responses. Perturbations of the epitranscriptome can lead to various pathogenic conditions, including cancer, cardiovascular abnormalities and neurological diseases. Recent advances in next-generation sequencing technologies have enabled us to identify and locate modified bases/sugars on different RNA species. These RNA modifications modulate the stability, transport and, most importantly, translation of RNA. In this review, we discuss the formation and functions of some frequently observed RNA modifications—including methylations of adenine and cytosine bases, and isomerization of uridine to pseudouridine—at various layers of RNA metabolism, together with their contributions to abnormal physiological conditions that can lead to various neurodevelopmental and neurological disorders.

## 1. Introduction

RNA is subjected to multifaceted regulatory processes to sustain diversity and complexity at the organismal and molecular levels. It has evolved to participate in diverse cellular processes owing to its capability to couple enzymatic activity with the storage and transfer of information. Upon being transcribed, the nascent RNA is subjected to various processing mechanisms, collectively termed post-transcriptional processing, that ultimately confer it with its information storage/transfer and regulatory functions. Post-transcriptional processing of eukaryotic RNA typically includes 5′ capping, intron removal or splicing, and addition of a 3′ polyadenylated tail. Another crucial mechanism of post-transcriptional RNA modification is the chemical modification of RNA bases and sugar residues on the RNA backbone. Similar to chemical modification of DNA cytosine residues that constitute part of the epigenome, chemical modifications of RNA, the “epitranscriptome”, adds another regulatory layer to organismal transcriptome-wide complexity. The functional impact of the epitranscriptome manifests in almost all tissues, but it is most apparent in regulating complex organs such as the brain. It is both transcriptomic and epitranscriptomic diversity that endows the nervous system with its complexity, with the latter altering various layers of RNA metabolism. RNA metabolism encompasses diverse processes including biogenesis, transport, splicing, stabilization, storage, and translation. Many recent studies have highlighted how dysregulation of RNA transport, splicing, stabilization, translation, or miRNA/tRNA biogenesis contributes to age-related neurodegenerative diseases [1] and neurodevelopmental disorders [2,3]. Precise spatial and temporal expression of various proteins is essential for appropriate brain development, which is achieved by proper accomplishment of RNA stabilization, transport and translation [4]. Even in the adult brain, RNA metabolism is one of the most crucial mechanisms for maintaining correct brain functions and learning-based memory consolidation [4]. Although different aspects of RNA metabolism contribute to neurodegenerative diseases and neurodevelopmental disorders, RNA-binding proteins (RBPs) play important roles in both kinds of disease pathogenesis [2,5].

According to the MODOMICS database, ~170 different RNA modifications have been identified to date, yet only a few have been well characterized and specifically linked to neurological disease [6]. RNA modifications are widely regarded as a regulatory tool for fine-tuning gene expression, acting beyond epigenomic regulatory mechanisms. Tremendous advancements in next-generation sequencing technologies have identified various RNA modifications at the single nucleotide level, which can be linked to mechanisms underlying tissue-specific and age-dependent gene expression profiles or to the pathophysiology of complex diseases such as cancer [5]. Functionally, three groups of protein factors—known as “writers”, “readers” and “erasers”—are involved in drafting the epitranscriptome, with writers and erasers possessing substrate-specific enzymatic activities. Together, these are known as RNA-modifying proteins (RMPs). Writers are the enzymes or effecters of RNA modification marks, whereas erasers are responsible for removing such marks. Readers recognize specific RNA modification marks, bind at that site, and activate downstream pathways, often altering RNA metabolism. Mutations and changes in the abundance of RMPs have been linked to various conditions, such as infertility and obesity, as well as neurodegenerative and neurodevelopmental diseases and cancer [7,8].

However, though RNA modification likely alters RNA metabolism, it remains enigmatic how such modifications contribute to neurodevelopmental and neurodegenerative diseases. In this review, we explore several exciting studies reporting the effects of different RNA modifications on various aspects of RNA metabolism and evaluate links between those modifications and a variety of neurological diseases.

## 2. RNA Metabolism-Associated Neurological Disease Mechanisms

### 2.1. mRNA Splicing

Introns of pre-mRNAs are removed and exons are joined in a process called pre-mRNA splicing to form mature mRNAs, and this process is regulated by several *cis*-acting elements and via formation of a multi-protein complex termed the spliceosome [9]. The involvement of different *cis*-acting elements alters exon recognition by spliceosomes, giving rise to alternatively spliced mRNAs from the same mRNA transcript. Alternative splicing not only contributes to diversity among species, but also enables tissue-specific expression of differentially spliced products to perform different functions [10]. mRNA splicing is emerging as a crucial mechanism for maintaining neuronal transcriptome complexity, shaping neuronal structure, function, and differentiation processes [10,11,12]. Perturbations of the essential association between *cis*-acting elements and splicing motifs result in splicing defects, potentially resulting in neurological disorders or neurodegenerative disease [13].

Effect on neurodevelopmental diseases: Approximately 1.4% of autism spectrum disorder (ASD) cases are caused by splicing defects [14]. Changes in splicing patterns of several mRNAs related to the PTEN signaling pathway have been observed in a murine model of ASD [15]. Animal models of neurodevelopmental disorders have also revealed alterations to the expression of position-dependent splicing factors. For example, neuro-oncologic ventral antigen (NOVA) and RNA-binding protein FOX (RBFOX) paralogs are positive regulators of exon inclusions during splicing of mRNAs linked to brain development, spine formation and neurite growth, and their downregulation has been observed in post-mortem brain tissue of autistic patients. Polypyrimidine tract-binding protein 1 (PTBP1) is a negative regulator of exon inclusion, and it is highly expressed during early embryonic development when it facilitates cells to enter the neuronal lineage. Abnormal (low) expression of this protein has been linked to schizophrenia-associated seizures [9].

Effect on neurodegenerative diseases: Alternative splicing also regulates the expression of different isoforms of α-synuclein, the main component of Lewy bodies and a hallmark of Parkinson’s disease (PD) [16]. Similarly, the ratio of alternatively spliced products of the *tau* gene product MAPT [13], namely 3R tau (formed upon exclusion of exon 10) and 4R tau (formed upon inclusion of exon 10) contribute to another well-known neurodegenerative disorder, Alzheimer’s disease (AD). Some recent studies have also highlighted alternative splicing and splicing defects as contributory mechanisms of different neurodegenerative diseases [17]. For instance, a TDP-43 mutation linked to ALS alters the splicing function of TDP-43, resulting in changed RNAs and contributing to early manifestation of the disease [18]. Splicing defects have been established as one of the major contributors for Huntington’s disease (HD) [19].CAG repeat expansion in SCA type 6-linked genes induce altered mRNA splicing patterns that result in accumulations of disease-causing polyglutamine-containing protein [20].

### 2.2. mRNA Alternative Polyadenylation

The alternative polyadenylation (APA) of mRNAs is the use of multiple polyadenylation sites in primary transcripts and in conjunction with alternative splicing. APA expands cellular transcriptomic diversity by generating distinct mRNA isoforms [21]. Depending on the location of polyadenylation sites (PASs), APA can be classified into two types: UTR-APA and coding region-APA (CR-APA) [22]. The presence of APA sites in 3′-UTRs of mRNAs generates transcript isoforms with the same coding region but with different lengths of 3′-UTR regions, thus giving rise to distinct interactions of mRNA isoforms with RNA-binding proteins and non-coding RNAs like microRNA and lncRNAs [21]. On the other hand, CR-APA directly affects the coding region and leads to the generation of proteins with different C-termini [23,24]. APA is found in all eukaryotes, and in mammals, about 70% of all mRNA-encoding genes undergo APA [25,26,27]. APA events can be tissue-specific to a great extent; for example, in the case of 3′-UTR APA isoforms, distal PASs are enriched in neurons, while blood cells and testis tissue favor the use of proximal PASs [28,29]. The functional consequences of APA sites in 3′-UTR of pre-mRNAs are diverse. For example, 3′-UTR-APAs participate in post-transcriptional gene regulation through various methods, such as modification of mRNA stability, translation, nuclear export and cellular localization. The influence of 3′-APA upon stability of mRNAs can be exemplified through altered effects of miRNA functions. For example, about 10% of all miRNAs targeting two cell types can be influenced by expression of APA isoforms [30]. Another way through which 3′-UTR APA events can modulate mRNA stability is differential binding of various RNA binding factors as well as lncRNAs that can affect the mRNA decay process [21]. The localization of mRNAs can also be influenced by 3′-UTR APA events, which is best exemplified in the case of *BDNF* transcripts, where the short isoform is restricted to the cell body while the long isoform is predominantly found in the dendrites [31]. Lastly, 3′-UTR APA events can directly influence protein localization, as evidenced in the case of proteins like CD47, CD44, α1 integrin (ITGA1) and TNF receptor superfamily member 13C (TNFRSF13C) [32]. CR-APA events are known to contribute to protein diversification, as seen in the case of transcripts encoded by genes like calcitonin-related polypeptide-α (*CALCA*) and immunoglobulin M heavy chain (*IgM*) [21]. CR-APA can also repress gene expression by generating severely truncated transcripts through utilization of PAS proximal to the promoter, as observed in the case of transcripts encoded by the mammalian polyadenylation factor cleavage stimulation factor 77 kDa subunit (*CstF-77*) gene [33].

Effect of neurodevelopmental diseases: Neuronal commitment at the early stages of neurodevelopment is heavily influenced by the transcriptome repertoire of neural stem cells. During neurodevelopment, APA contributes significantly to the specification of neuronal lineage in association with other mechanisms such as microRNA networks, alternative splicing, non-sense mediated RNA decay, etc., that shape the transcriptome diversity of neural stem cells. APA events are known to be enriched in specific neuronal cell types [34,35]. Additionally, single-cell RNA sequencing data analysis identified cell type-specific APA landscapes in different GABAergic interneurons in the mouse cerebral cortex. Interestingly, genes with cell type-specific APA events are enriched in biological processes like synaptic vesicle recycling, neurotransmitter release, ion transport etc., which implies a significant role of APA in synaptic communication and neuronal identity determination [36]. Furthermore, the role of APA during early stages of neurodevelopment, such as the commitment and differentiation of neural progenitors, has been investigated by Grassi et al. where transcriptome-wide changes of 3′-UTR lengths were observed during differentiation of mouse-adherent neural stem cells into GABAergic inhibitory neurons [37]. A group of studies have linked APA events and 3′-UTR in specific genes like *MeCP2*, *FMR1* to disorders with autistic phenotypes such as Rett syndrome, Fragile X-associated syndrome, autism, schizophrenia and other psychiatric diseases [38,39,40,41,42]. Since ASDs have been correlated with aberrations of calcium signaling, the dysregulation of APA events in the autistic brains, as found by analyzing RNA sequencing data from publicly available databases, are linked with dysregulation of calcium ion homeostasis by Szkop et al. [43]. The effect of APA in the regulation of MeCP2 protein levels and concomitant development of neuropsychiatric diseases has been studied by Gennarino et al., where copy-number variation of the *NUDT21* gene that encodes a subunit of pre-mRNA cleavage factor Im is reported to regulate the length of *MeCP2* transcript 3′-UTR [44].

Effect of neurodegenerative diseases: The ability of APA events to generate transcripts with varying lengths of 3′-UTR gives rise to their intimate association with the regulation of gene expression. Since significant alterations of gene expression have been observed in neurodegenerative disorders [45,46], APA can be viewed as a potentially important regulatory mechanism operating during the development and progression of different neurodegenerative diseases. Analysis of RNA sequencing data from AD, PD and ALS patients and matched healthy controls, available in public databases, revealed disease-specific changes of APA profiles in a subset of genes among each disease [47]. Although this study found APA profile changes in relatively small subset of genes, and affected genes differ among RNA-sequencing datasets, they found, in all three disease-associated datasets, overrepresentation of genes associated with protein turnover and mitochondrial function. Usage of the distal PAS site in α-synuclein mRNA generates an extended transcript isoform which is shown to be associated with PD development, and the presence of this extended 3′-UTR promotes accumulation of the α-synuclein protein, which gets redirected away from the synaptic terminal towards mitochondria [48]. Genome-wide usage of proximal PAS within 3′-UTR regions or PAS within introns leads to transcriptome-wide shortening of 3′-UTR regions, and that may underlie the development of neurological disorders like oculopharyngeal muscular dystrophy (OPMD) [49]. The effect of APA events on the regulation of the localization and stability of mRNAs is conclusively exemplified in the case of mRNAs encoded by the gene *huntingtin* (*Htt*), involved in the development of HD. The *huntingtin* mRNA has two isoforms differing in the length of 3′-UTR. The abundance of *Htt* isoforms differs among cerebellum, motor cortex, fibroblasts and neural stem cells from patients and controls. Additionally, two isoforms differ with respect to their localization, the length of their poly-A tail, their half-lives and their binding sites for miRNA- and RNA-binding proteins. Moreover, the mRNA 3′-UTR isoform change is not restricted to *Htt*; 11% of alternatively polyadenylated genes in the HD motor cortex undergo changes with respect to the abundance of their mRNA isoforms [50]. Reduced expression of TDP-43 has been associated with the utilization of a intronic cryptic polyadenylation site of the *stathmin-2* gene in ALS and frontotemporal dementia (FTD). This results in reduced expression of the *stathmin-2* gene, which is a hallmark of ALS-FTD [51]. Finally, alternative polyadenylation of human *tau* gene has been associated with binding of mir-34 family members of miRNAs and expression of *tau* mRNA isoforms [52].

### 2.3. mRNA Transport and Translation

Owing to the presence of extended neuronal processes, such as long axons and dendrites, it requires more energy and time to transport proteins on demand from the soma to distal parts of neurons. However, mRNAs are transported along neurites together with ribosomes and all the translation machineries, so mRNAs are ready to be translated in different parts of neurons [53]. Recent investigations have found that ribosomes are assembled at the distal end of axons instead of being formed from proximally translated ribosomal proteins and transported as part of mRNP complexes to the distal site [54,55]. Moreover, various mRNAs can be transported together, yet remain translationally repressed. RBPs play important roles in both mRNA transport and translational repression. Dysregulation of dendritic mRNA transport/translation causes aberrant spine formation and dendritic structural anomalies, as well as learning memory impairments, that are symptoms of neurodevelopmental disorders [56]. Axonal transport and translation of mRNAs are required to maintain the structure of axons and their function in conducting nerve impulses [57]. Clearly, if axonal mRNA transport/translation is impaired, neuronal functions will be severely compromised, diminishing neuronal survival, as observed for many neurodegenerative diseases [58]. A new mode of translation dysregulation has come to surface through the recent discovery of repeat-associated non-AUG (RAN) translation [59]. This mode of translation gets activated due to tandem repeat expansion beyond the threshold during repeat expansion diseases and causes translation from all three reading frames. These mis-translated proteins are accumulated in diseased tissues to manifest disease phenotypes [60].

Effect on neurodevelopmental diseases: An impressive body of work has uncovered how translational dysregulation of mRNAs is linked to ASD and Fragile X syndrome (FXS) [61]. Most of the experimentally-validated mRNAs (e.g., *Map1b*, *GluR1*, *Rac1*, *CamKII*, *Shank3*, *Gabrb1*, among others) are targets of the RBP Fragile X mental retardation protein (FMRP) and are associated with synaptic structural anomalies and dysfunction, as well as impairments of long-term memory formation [61,62,63]. Furthermore, genetic mutations of several core translation regulatory proteins, e.g., RPL10, eIF4E, UPF3B, GW182, CYFIP1, Caprin1, eIF2B, and PTEN, have also been linked to ASD and other neurodevelopmental disorders such as infantile epilepsy, mental retardation, schizophrenia, attention deficit hyperactivity disorder (ADHD) and many more. More than 1000 such genes have been included in the Simons Foundation Autism Research Initiative (SFARI) database (https://gene.sfari.org/; accessed date July 2021). Further research is in progress to establish the molecular mechanisms underlying translational dysregulation of the mRNA targets of these proteins [64].

Effect on neurodegenerative diseases: Patients suffering spinal muscular atrophy (SMA) exhibit reduced binding of survival motor neurons (SMN) to small nuclear RNA (snRNAs) because of genetic mutation-driven impairment of SMN protein stability, resulting in abnormal snRNA trafficking and maturation [65]. In contrast, ALS-linked mutations enhance stress granule formation or cause aberrant clearance, resulting in larger RNA-protein assemblies [66]. These examples indicate that either hyper- or hypo-assembly of mRNPs causing aberrant transport of mRNAs can lead to many neurodegenerative diseases [65]. Atypical transport/translation of mRNAs associated with the muscleblind-like (MBLN) group of proteins causes myotonic dystrophy (DM) [67]. RAN translation in the *c9orf72* gene harboring G4C2 repeat expansion mutations at intron 1 has been established as the main cause of ALS and FTLD diseases [68]. In SCA31, expansion of a TGGAA repeat in the BEAN1 transcript causes accumulation of pentapeptide repeat protein translated from all three reading frames using a similar mechanism. Moreover, a UGGAA repeat containing an abnormally structured RNA, known as an RNA foci, sequesters RBPs, affecting their functions and thus contributing to disease phenotypes [69]. RNA foci and the activation of RAN translation are also implicated in SCA8, HD and many other triplet repeat disorders [70,71]. Recently, mutant huntingtin protein was shown to stall ribosomes, thereby affecting the translation of several mRNAs (including *Mfsd10* and *Ppbp*) that contribute to HD progression [72]. Deviant axonal transport of mRNAs associated with TDP-43 (*Map1b*, *Nefl*) or with FUS (e.g., *Fosb*) contributes to ALS and frontotemporal lobar degeneration (FTLD) [73,74]. Interestingly, translational activation of CyclinD1 and TDP-43 mRNAs via Ataxin2-mediated polyadenylation in association with the Poly-A binding protein PAPD4 can induce TDP-43 proteinopathies, such as the Tau aggregation typical of FTLD, ALS, and AD [75,76]. Together, this evidence establishes dysregulated mRNA transport/translation as a crucial factor in several neurological diseases.

### 2.4. mRNA Stability

To maintain RNA homeostasis, mRNAs transcribed inside the nucleus decay through various biological processes directed by *cis*-acting elements. Exonucleases and endonucleases contribute to these decay processes [77]. Methylation capping at the 5′ untranslated region (UTR) and polyadenylation at the 3′-UTR protect mRNAs from degradation by these nucleases. Gene expression levels are dependent on mRNA stability, which is measured by the half-lives of mRNAs [78]. mRNA half-life can be increased or decreased by diverse mechanisms [79]. Alternatively spliced mRNAs can harbor or exclude *cis*-acting elements or enable alternative polyadenylation, thereby regulating the stability of the mRNA [80].

Effect on neurodevelopmental diseases: The Hu/Elav group of proteins exert an important role in exon inclusion and differential polyadenylation to alter the stability of mRNAs such as *Bdnf* and *Nf1*, thus regulating neuronal differentiation and function [81]. HuD-null mice exhibit sensory and motor neuron defects [82]. Moreover, neuronal Elav-like (nELAVL) protein has been associated with ASD [83]. Reduced expression of the mRNA stability-related protein RBFOX1 has also been linked to ASD [4]. Recent experimental evidence has further confirmed that FMRP can alter ASD-related mRNA stability to counter Ataxin2-mediated changes in gene expression under different kinds of cellular stress [84].

Effect on neurodegenerative diseases: nELAVL-mediated changes in mRNA stability have also been implicated in neurodegenerative diseases such as AD and PD [85]. A recent study reported that Ataxin2 endows stability on its mRNA target TDP-43, with this function being dependent on its poly-Q domain. Expansion of the poly-Q domain of Ataxin2 alters TDP-43 mRNA stability, resulting in tau protein aggregation and ALS pathogenesis [86]. Another RBP, RBFOX, stabilizes mRNAs encoding synaptic transmissions, and its dysregulation has been linked to AD [87]. Proteins primarily known to regulate other forms of RNA metabolism are also known to alter RNA stability. For instance, TDP-43 participates in stabilizing β-adducin (Add2) mRNA. This phenomenon is predicted to be associated with ALS and FTLD diseases, though its exact mechanism is not yet understood [88]. Thus, different RBPs work together to maintain mRNA/protein homeostasis in the cell by changing mRNA stability and translation. Any failure in this coordinated effort can induce neurological pathogenicity.

### 2.5. miRNA Biogenesis

Micro-RNAs (miRNAs) are small non-coding regulatory RNAs that post-transcriptionally silence specific mRNAs, representing another form of temporal gene expression control. These miRNAs are involved in fine-tuning gene expression required for neural development, structure and function, so aberrant miRNA activity can induce neurological disease [89]. miRNA profiling has revealed that a considerable number of miRNAs are expressed in the hippocampus of the adult brain in an activity-dependent manner. For instance, miR-132 is expressed under KCl- or DHPG-driven neural activation, and miR-212 is regulated via the CREB activation pathway [90,91].

Effect on neurodevelopmental disease: miRNA biogenesis has been implicated in synaptic plasticity and long-term memory formation [89]. Dysregulated miRNA synthesis and maturation contribute to ASD, intellectual disability, and schizophrenia [92].

Effect on neurodegenerative diseases: Interestingly, the progression of neurodegenerative diseases also appears to be dependent on the differential expression of miRNAs. Post-mortem AD brains display significantly different miRNA expression profiles compared to age-matched controls [93,94]. Specifically, reduced expression of miR-9 in the hippocampus and miR-107 in the cortex were observed in AD brains, and this feature was linked to aberrant expression of BACE1, Sirtuin1, and PSEN1. In contrast, miR-7, miR-153, miR-34b, miR-224, and miR-379 regulate accumulation and aggregation of α-synuclein, a hallmark of PD [95]. ALS-linked inflammation has been linked to dysregulation of miR-577, miR-155, and let-7 [96]. Moreover, miRNA expression and functions may also be partially responsible for other neurodegenerative diseases such as HD and MD [97,98].

Different RNA metabolisms described above are also shown in Table 1.

### 2.6. Roles for RBPs in RNA Metabolism and Neurological Diseases

Various mechanisms of RNA metabolism temporally regulate the protein expression responsible for brain development, structure and function. RBPs fine-tune RNA metabolism, resulting in further complexities of gene expression in different neuronal parts [5]. Expression-mediated RBP functions may be countered by other RBPs, thereby maintaining a balance of RNA metabolism and gene expression for neurons in different parts of the brain. Dysregulated RBP functioning and enrichment can severely perturb such control mechanisms, resulting in neurodevelopmental or neurodegenerative diseases (summarized in Table 1) [111,112].

## 3. RNA Modifications that Change RNA Metabolic Processes

**Figure 1:**
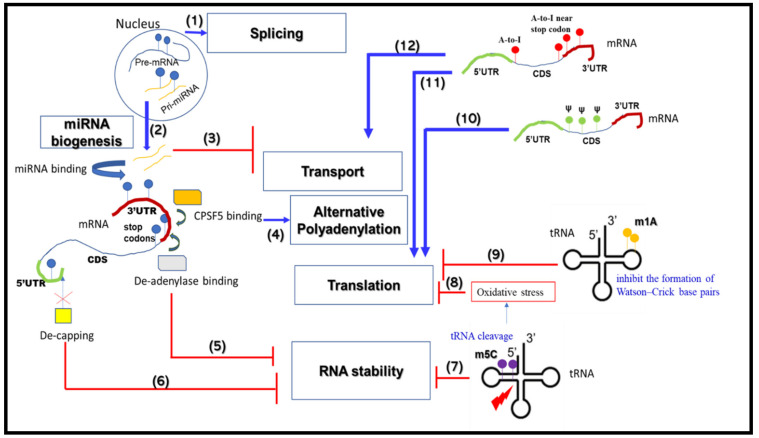
Illustrative model summarizing how various RNA metabolic processes are modulated by RNA modifications. Different RNA modifications, e.g., m6A, m5C, m1A, A-to-I RNA editing and pseudouridine, are represented by blue, purple, yellow, red and yellow colored pins, respectively. Various mechanisms of activation or inhibition of RNA metabolisms by RNA modifications are represented by (**1**) to (**12**), e.g., m6A modifications of pre-mRNAs (**1**) and miRNAs (**2**) facilitate splicing and miRNA biogenesis, respectively. The model shows that m6A modification of the 3′-UTR of mRNAs facilitates binding of miRNAs to this region and inhibits mRNA transport/translation (**3**). VERMA-mediated m6A modification near the 3′-UTR and stop codons of mRNAs facilitates alternative polyadenylation (**4**). Alternatively, m6A modification near the 3′-UTR and stop codons causes de-adenylase binding, thereby impairing stability (**5**). De-capping at the 5′-cap site with nearby m6A inhibits translation initiation and also reduces mRNA stability as a result of endonuclease activity (**6**). m5C modification of tRNAs induces their cleavage, thus altering RNA stability (**7**). Accumulations of cleaved tRNA fragments induce oxidative stress, which inhibits cellular translation (**8**). m1A modification impairs base pairing of tRNA-anticodons with the mRNA initiation codon, inhibiting translation initiation (**9**). Both A-to-I editing and pseudouridine modification alter start or stop codons of mRNAs, blocking mRNA transport/translation (**10**, **11**, **12**).

Despite hundreds of RNA modifications on coding and non-coding RNAs having been identified to date, only a few have been studied extensively or linked to disease. Well-studied RNA modifications include the methylation of adenosine at position 6 (m6A, also known as N6-methyladenosine), N1-methyladenosine (m1A), 5-methyl cytosine (m5C), pseudouridine, and RNA editing, e.g., A-to-I (see Figure 1) [113,114].

### 3.1. m6A

m6A is the most studied RNA modification in humans. It is a dynamic and reversible modification of RNA. m6A marks have been identified on mRNAs, transfer RNAs (tRNAs), ribosomal RNAs (rRNAs), non-coding RNAs (ncRNAs), circular RNAs (circRNAs), and miRNAs. On mRNAs, most m6A marks are found at the beginning of the last exons, in the 3′-UTR or near the stop codons. Methyltransferase complexes, such as METTLE3, METTLE4 and WTAP, act as writers to methylate adenosine at position 6. Demethylases such as FTO and ALKBH5 are the known erasers of m6A modification marks [115,116]. There are three different groups of readers that recognize m6A marks on RNAs and can alter patterns of RNA metabolism. The first group comprises heterogeneous nuclear ribonucleoprotein A2/B1 (HNRNPA2B1), which regulates splicing and miRNA processing by binding to m6A marks on certain miRNAs [117]. The second group is YTH-RNA binding domain-containing proteins, which regulate splicing, mRNA stability, translation and miRNA synthesis [114]. The third group includes insulin-like growth factor 2 mRNA-binding proteins (IGF2BPs) that recognize m6A marks in the 3′-UTRs of mRNAs and enhance mRNA stability [118,119].

m6A modifications impact dendritic structure, spinogenesis, learning memory, neurogenesis, axon regeneration and brain development [115,120]. Neurological disease phenotypes arise from dysregulated m6A pathways, owing to disease-specific mutations or changes in amounts of various m6A players. Although the underlying mechanisms remain incompletely understood, RNA modifications at m6A positions have been implicated as contributing to epilepsy, intellectual disability, and schizophrenia [121]. m6A modification also plays a very important role in AD by altering the protein levels of transcripts responsible for disease phenotypes [122]. The m6A modifications at the 3′-UTR of mRNAs associated with age related disorders, e.g., AD, PD, FTLD etc., have been thought to play important mechanistic roles in manifestation of these diseases, mostly through modulation of translation of these transcripts [116,123]. Dysregulated m6A modification in other RNA metabolic processes, such as splicing, has also been implicated in neurodegenerative diseases [124]. In Table 2, we present m6A modifications and changes in RNA metabolism linked to neurological diseases.

### 3.2. m1A

Methylation of the N1 position of adenosine is another dynamic RNA modification in mammalian systems. It stalls translation elongation and prevents misincorporation of nucleotides during reverse transcription (RT) by inhibiting the formation of Watson–Crick base pairs [125]. M1A modifications primarily occur in tRNAs and rRNAs, changing their topology and modulating their protein-binding ability, stability and functioning [126]. m1A modifications have also been reported in the 5′-UTR (where they accelerate translation) and coding sequence (CDS, where they inhibit translation) of mRNAs [127]. The most abundant cytosolic m1A writer is the hetero-tetrameric tRNA methyltransferase TRMT6/61A. The TRMT6/61A complex adds a GUUCRA tRNA-like motif with a t-loop structure to targeted nuclear mRNAs [128]. In contrast, the homodimeric TRMT61B methyltransferase acts as an m1A writer of mitochondrial mRNAs [129]. TRMT10C, another m1A methyltransferase, is responsible for modifying the mitochondrial mRNA ND5 [130]. ALKBH3 and ALKBH1 are m1A erasers, with ALKBH3 acting to demethylate the m1A mark on mRNAs [131]. As for other RNA modifications, m1A readers play important roles in regulating RNA metabolism, triggering tissue-specific and time-dependent functions. Nine putative m1A readers have recently been identified, including ribonucleoproteins and YTH family proteins such as YTHDF3 [132]. Links between m1A marks and neurological diseases have not been well established. m1A mRNA modifications are mainly associated with modulating mRNA translation and decay, thereby likely playing important roles in appropriate brain development and function [133]. A recent study associated lack of m1A marking in mitochondrial tRNA with the rare neurodegenerative disease HSD10 [134].

**Table 2:** Effects of RNA modifications on various RNA metabolic processes and their association with neurological functions and diseases.

RNA Modification	Effect on RNA Metabolism and/or Protein Function	Reader/Writer/Eraser	Mechanism	Neurological Functions/Diseases	References
**m6A**	mRNA stability	Writer: METTL3, METTL14 Reader: YTHDF2, YTHDF3, YTHDC2	Readers selectively recognize and bind G-(m6 A)-C-containing mRNAs via their CTD, whereas the NTD localizes mRNP complexes at the cellular RNA degradation machinery	Neurogenesis	[135]
	Splicing, Transport	Reader: YTHDC1	In the nucleus, this reader recognizes and binds pre-mRNAs with m6A methylation marks and selectively recruits SRSF3 to promote exon inclusion and nuclear-to-cytoplasmic transport of target mRNAs.	Facilitates neuron survival after brain injury and ischemic stroke	[120]
	Transport, Translation	Reader: YTHDF1	This reader recruits the mRNP complex to the cellular transport and translation machinery and activates protein translation	Facilitates learning and memory development	[116]
	Translation	Eraser: FTO	In diseased neurons, FTO is translated and accumulates at axons, increasing m6A demethylation and NMDAR1 expression followed by neuronal apoptosis	PD	[115]
	Translation	Writer: METTL3 Eraser: FTO	mRNA methylation controls expression of AD-related transcripts, but the underlying mechanism remains obscure	AD	[122]
**m1A**	tRNA stability/Translation	Writer: MRPP1	Mitochondrial tRNA methylation causes stabilization of the tRNA to facilitate translational initiation. In disease conditions, improper processing of tRNAs results in reduced mitochondrial protein synthesis	HSD10 disease	[134]
**m5C**	Translation	Writer: NSUN2	In the absence of tRNA methylation, angiogenin-mediated tRNA cleavage causes an accumulation of tRNA fragments that activate stress-response pathways and impair translation	Dubowitz-like syndrome, Noonan like syndrome	[133]
	tRNA stability/Translation	Writer: DNMT2	Methylation of tRNAs enhance their stability and facilitate their translation tRNA cleavage as a result of impaired m5C methylation limits translation in diseased neurons	Brain development and neurogenesis, embryogenesis	[136,137,138]
**Pseudoeuridine**	mRNA stability, Translation	Writer: PUS1	Exact mechanism not yet known. It is likely the presence of pseudoeuridine reduces mRNA stability and impairs translation	AD	[139]
**RNA editing**	Transport/Translation	Writer: ADAR2	AMPA receptor pre-mRNA is edited by ADAR2 to regulate its function. Downregulation of ADAR2 causes reduced editing accompanied with functional defects of AMPAR under disease conditions	Schizophrenia, mood disorders	[140,141]

CTD—C-terminal domain; NTD—N-terminal domain.

### 3.3. m5C

Cytosine carbon-5 methylation (m5C) of DNA has long been considered a highly important epigenetic modification impacting diverse physiological processes such as cellular differentiation and organism development, and it has been implicated in cancers and cardiovascular abnormalities. In more recent years, m5C modification of RNAs has gained increasing attention owing to its roles in RNA transport, translation and stability [142,143,144]. m5C modifications have been reported for rRNAs, tRNAs, and mRNAs, as well as less abundant RNA species such as vault RNAs, small nuclear RNAs (snRNAs), enhancer RNAs, long non-coding RNAs, and miRNAs [142,145,146,147]. In humans, the NOL1/NOP2/SUN domain-containing family of proteins (NSUN1-7) and the DNA-methyltransferase (DNMT) homologue DNMT2 create m5C marks [148]. NSUN1, NSUN2 and NSUN5 are conserved in all eukaryotes, but other family members are only found in higher eukaryotes. Functionally, NSUN1 and NSUN5 methylate cytosine C5 of cytosolic rRNAs, whereas NSUN2, NSUN6 and DNMT2 methylate cytosolic tRNAs. NSUN2, NSUN3 and NSUN4 methylate mitochondrial RNAs at cytosine C5 [149,150]. NSUN2 can also m5C-methylate mRNAs [142]. NSUN7 regulates expression levels of target genes of the transcriptional co-activator PGC-1α by regulating the stability of their respective enhancer RNAs [151]. NSUN proteins and DNMT2 are both SAM-dependent methyltransferases. Mechanistically, they differ in terms of the number of catalytic cysteines they use; whereas NSUN proteins use two catalytic cysteines, DNMT2 uses a single catalytic cysteine at the active site [152,153]. Aly/REF export factor (ALYREF) and Y-box binding protein 1 (YBX1) recognize and bind to m5C, and these reader proteins are involved in nucleo-cytoplasmic shuttling and stability of mRNAs [142,144]. As for C5-methylated cytosine residues in DNA, m5C modification of RNAs is also highly dynamic and can be actively demethylated by Fe (II)- and α-ketoglutarate (α-KG)-dependent dioxygenases such as TET1 and TET2 [154].

m5C RNA methylation impacts diverse RNA metabolic processes. For instance, eukaryotic rRNA stability is affected by the presence of m5C marks, influencing folding of essential ribosomal regions and thereby regulating translation [155]. m5C modification of tRNAs alters their structure and stability, and thus translation, as also observed for C34 methylation of yeast tRNA^Leu(CAA)^ [136]. Moreover, m5C modification can affect the aminoacylation step of translation and, consequently, overall translational accuracy [137,156]. Recent studies of *Arabidopsis thaliana* and zebrafish have shown that mRNA stability is regulated by m5C modification [144,157]. In a HeLa cell line, NSUN2-mediated m5C modification of mRNAs was found to enable their binding with ALYREF, an mRNA transport adaptor that facilitates nuclear export [142]. Interestingly, the effects of m5C modification on mRNA translation vary depending on the site of m5C deposition; it impairs translation efficiency when it occurs in the 5′-UTR or CDS [143,157,158], yet NSUN2-mediated m5C modification in the 3′-UTR enhances translation efficiency [159].

The crucial functional roles played by m5C methyltransferases in RNA metabolism imply that impaired expression of these genes may be responsible for various diseases. For example, several neurodevelopmental disorders have been linked to mutations in genes encoding NSUN proteins [160]. Deletion of NSUN2 from mice induces growth retardation and delayed/blocked tissue-specific differentiation [161,162]. In humans, a homozygous mutation of the *NSUN2* gene that causes a non-synonymous substitution of glycine at position 679 with arginine results in mislocalization of the protein in the nucleolus, which has been linked to the development of autosomal-recessive intellectual disability [163,164]. Similarly, Dubowitz syndrome—a rare autosomal recessive disorder that clinically manifests as microcephaly, abnormal facial phenotypes, mental retardation and short stature—has been linked to a homozygous mutation in the splice acceptor site of *NSUN2* exon 6 that reduces mRNA stability and NSUN2 protein levels, resulting in diminished methylation of target RNAs [165]. A recent study investigating conditional disruption of *NSUN2* in the prefrontal cortex of mice observed a bidirectional change in depression-related behaviors. NSUN2 deficiency resulted in changes in 1488 proteins in the prefrontal cortex, together with a decline in translation efficiency associated with a glycine-codon defect. Consequently, the mice displayed impaired synaptic signaling of pyramidal neurons in the prefrontal cortex and defective contextual fear memory [166]. Disruption of NSUN2-mediated tRNA methylation causes 5′ regions of tRNA fragments to accumulate, impairing the generation of upper layer neurons and brain development in mice [167]. Furthermore, a loss-of-function mutation in *NSUN3* has been identified in a patient suffering severe mitochondrial respiratory chain complex deficiency, characterized by combined developmental disability, microcephaly, recurrent increased lactate levels in plasma, and muscular weakness [168].

### 3.4. Pseudouridine (Ψ)

Pseudouridine is a uridine isomer, and it is mostly found in non-coding RNAs such as rRNAs, tRNAs, and snRNAs. High-throughput sequencing has revealed the Ψ modification in yeast and human mRNAs [169]. Two distinct mechanisms of RNA pseudouridylation have been proposed. Firstly, guide RNA-dependent pseudouridylation involves H/ACA-box small nucleolar RNAs (snoRNAs) that bind to target RNAs via sequence-specific interactions, followed by catalytic uridine modification by specific enzymes (such as dyskerin in human or Cbf5 in yeast) present in the H/ACA-box snoRNA riboneucleoprotein (snoRNP) complex. Secondly, guide RNA-independent pseudouridylation requires pseudouridine synthase (PUS) enzymes that directly catalyze conversion of uridine to Ψ in their targets without any accessory RNA contribution [169,170,171]. PUS proteins are conserved from yeast to human and have been classified into six families. Isomerization of uridine to Ψ favors base stacking, thus resulting in enhanced stability of RNA secondary structures. Moreover, the presence of Ψ in several RNAs alters their interaction with RBPs that function in nuclear RNA processing and cytosolic RNA localization or stability [169,170]. Ψ modification also affects biogenesis of rRNAs, tRNAs, tRNA-derived small RNAs and snRNAs. Pseudouridylation of yeast and human mRNAs is regulated under stress conditions such as serum deprivation, H_2_O_2_ treatment and heat shock.

Several studies have illuminated the impact of pseudouridylation on neuronal functions. For instance, nociceptor neuron-specific loss of *RluA-1* and *RluA-2*, which encode *Drosophila* PUS enzymes, causes hypersensitive nociception phenotypes such as thermal hyperalgesia [172]. Elevated amounts of Ψ have been identified in the urine of AD patients, though this feature has not been symptomatically linked to AD [173]. deLonimier et al. have shown that Ψ modification of the expanded intronic CCUG repeat in *CNBP*, expression of which is associated with type 2 myotonic dystrophy (DM2) in humans, results in reduced binding of muscleblind-like 1 protein to CCUG repeat-expanded *CNBP* RNA linked to DM2 pathogenicity [174]. Thus, pseudouridylation can be regarded as a therapeutic target for neurodegenerative diseases such as DM2. Abnormalities in *PSU* genes are known to cause neuronal dysfunction. For example, two point mutations in *PUS1* were identified in a patient presenting with mild cognitive impairment and sideroblastic anemia since childhood, together with adult-onset hepatopathy, cardiomyopathy and insulin-dependent diabetes, all of which are typical clinical signs of myopathy-lactic acidosis-sideroblastic anemia (MLASA) syndrome [175]. Furthermore, observations of *Pus3* mRNA expression in the nervous system of mouse embryos indicates a possible role for it in neuronal development, which is corroborated by a report linking PUS3 truncation to intellectual disability in human [176,177]. Mutations in *PUS7* have been identified in human patients displaying intellectual disability, microcephaly, speech delay and aggressive behavior [178,179]. These mutations limit Ψ modification at position 13 of tRNAs and reductions in PUS7-targeted mRNAs, with these phenotypes being recapitulated in Drosophila upon *PUS7* knockout [178]. Moreover, higher expression levels of *Dyskerin 1* (*Dkc1*) coding for a PUS component of the H/ACA-box snoRNP complex were observed in mouse embryonic neural tissues and specific neurons of the cerebellum and olfactory bulb of adult mouse brain, implying a potential role for it in neurodevelopment [180].

### 3.5. RNA Editing

RNA editing is a type of modification whereby adenosine (A) or cytosine (C) residues are converted to inosine (I) or uridine (U) residues, respectively. The C-to-U conversion was first noted in the mRNAs of mitochondrial *cytochrome c oxidase subunit II* (*COX-2*) of trypanosomes. In mammalian systems, it was first reported in the mRNA of *hepatic apolipoprotein B*, where it alters glutamate residue 2153 to a stop codon and results in formation of a truncated apolipoprotein B isoform known as Apo-B48 [181]. RNA editing is now known to be conserved from Drosophila to humans, and even operates in bacteria [182,183,184,185]. Most of the editing happens in the repetitive elements, such as primate-specific Alu repeats within introns or untranslated regions [186]. Besides that, a small number of editing events happen in coding regions or microRNA sequences [187,188,189]. A-to-I editing is catalyzed by adenosine deaminases (known as ADARs for Adenosine Deaminases Acting on RNA). Mammals have four ADARs—ADAR1p150, ADAR1p110, ADAR2 and ADAR3—generated from three *ADAR* genes (*ADAR1-3*), whereas *Caenorhabditis elegans* has two *ADAR* genes and Drosophila has only one [190,191]. Deamination of adenosines in tRNAs is accomplished by ADATs (Adenosine Deaminases Acting on tRNA). Eukaryotes are reported to have three *ADAT* genes (*ADAT1-3*) [192]. *Escherichia coli* too has a tRNA-specific deaminase [193]. C-to-U editing is catalyzed by activation-induced cytidine deaminases/apolipoprotein B-editing complex (AID/APOBEC) [194,195]. The AID/APOBEC family of deaminases (AADs) in humans has 11 members. AADs are thought to have evolved from bacteria following lateral gene transfer and then diversified [196,197].

RNA editing exerts multiple functional impacts on RNA metabolism. A-to-I and C-to-U editing both influence RNA secondary structure [198,199]. RNA editing impacts gene expression by modulating the expression, processing or stability of miRNAs [200,201,202]. Importantly, A-to-I RNA editing is known to influence epitranscriptomic and proteomic diversity in cancers [203,204].

RNA editing has a myriad of effects on brain functioning. A prominent role for RNA editing has been observed in the case of AMPA and Kainate glutamate receptors, whereby the glutamines (Q) of Q/R sites are replaced by arginine (R). This change leads to Ca^2+^ impermeability and has been linked to the formation of long-term potentiation in synaptic plasticity. Absence of GluA2-Q/R editing results in epileptic seizures and death in mice due to uncontrolled Ca^2+^ influx [205]. A role for GluA2-Q/R editing in embryogenesis and in vitro differentiation of neural progenitor cells has also been reported [206]. Moreover, RNA editing has been linked to altered neuronal signal transduction, whereby editing at five closely-spaced but different sites of 5-HT_2C_ serotonin receptor transcripts in human and rodents reduced the efficiency of G-protein coupling [206]. Evidence for RNA editing contributing to the generation of neuronal diversity in the fly brain comes from data showing ample editing events in the highly conserved regions of transcripts encoding channel proteins and other essential neuronal proteins [207]. This form of RNA modification also influences subunit assembly of the excitatory AMPA glutamate receptor and inhibitory GABA_A_ receptor [206], and it regulates the activity of neuron-specific transcription factors such as glioma-associated oncogene 1 (GLI1) and Nova1 [208,209].

In humans, approximately 85% of all pre-mRNAs undergo A-to-I RNA editing [186,210,211]. This RNA modification underlies many neurological and neurodegenerative disorders. As stated above, epilepsy and related diseases are caused by dysregulated RNA editing of AMPA receptors. Similarly, ADAR2-mediated I/V site RNA editing of the potassium channel Kv1.1 has been linked to epilepsy. Mutations in *KCNA1*, which encodes Kv1.1, cause episodic ataxia type 1 (EA1), typically characterized by seizures, myokymia and ataxia. GluA2-Q/R site editing was found to be reduced in the motor neurons of ALS patients, which enhances Ca^2+^ influx and thereby elevates calpain activation to cleave more TDP-43, which is associated with mis-localization and cytoplasmic aggregation of TDP-43, a hallmark of ALS [212]. GluA2-Q/R editing efficiency at 30 different sites is reduced in the brains of AD patients [213]. Moreover, cerebral ischemia of CA1 pyramidal neurons significantly reduces RNA editing of AMPA receptors [212]. Psychiatric diseases such as schizophrenia and mood disorders have been linked to ADAR2-associated RNA editing of glutamate receptors [140] (see Table 2), and dysregulated editing of *Htr2c* mRNA (encoding serotonin receptor subtype 5-HT_2C_R) may increase the propensity for suicide [212]. Notably, FMRP protein, the loss of which is responsible for FXS onset, regulates RNA editing by binding to the ADAR proteins of Drosophila [214], zebrafish [215], mice [216] and in human cell cultures [217]. Lastly an interesting report by Kawahara et al. related the effect of the editing of miRNA with an X-chromosome-linked human disorder characterized by gout and neurodevelopmental impairment with hyperuricemia. Their study revealed that tissue-specific A to I editing of miR-376 cluster transcripts resulted in targeting of a distinct set of genes by edited miRNA. Repression of one of the edited mature-miR-376 target genes, namely, phophoribosyl pyrophosphate synthetase 1, was implicated in tissue-specific regulation of uric acid levels [201]. Therefore, overall, RNA editing has been linked to diverse neurodevelopmental disorders such as Prader-Willi syndrome, ASD, FXS, and others.

## 4. Conclusions

With the rise of epitranscriptomics, another level of gene expression control beyond transcription, translation, and epigenetic regulation has emerged as one of the most interesting fields of research. Several studies have now shown that post-transcriptional RNA modifications play a significant role in the spatio-temporal expression of diverse proteins in the brain. Neural development from embryonic to adult stages, neurogenesis, and the maintenance of proper neuronal structure and functioning necessitate tightly controlled protein expression. RNA modification writers and erasers control the presence or absence of epitranscriptional marks such as m6A, m1A, m5C, Ψ and RNA editing on diverse RNAs. Readers interpret these modifications to recruit RBPs that modulate RNA metabolic pathways, including splicing, mRNA decay, miRNA biogenesis, alternative polyadenylation, transport, and translation. In this way, RNA-modifying regulatory proteins and RBPs orchestrate the finely-tuned gene expression landscape to ensure appropriate brain development, structure and functioning. In neurodevelopmentally- and neurodegeneratively-diseased brains, these control mechanisms fail, likely due to disease-specific mutations or abnormal aggregates that trap epitranscriptomic proteins. More extensive studies are now being undertaken in this field that will enrich our understanding of how RNA modifications and their regulatory proteins change under conditions of neurological/neurodegenerative disease.

## Figures and Tables

**Table 1:** Dysregulated RNA metabolism in neurological diseases.

Disease Type	Altered RNA Metabolism Pathway	RBP(s) Involved	Mechanisms	Neurological Disease(s)	References
Neuro developmental diseases	Splicing, Translation	CPEB4	Missplicing of *CPEB4* causes reduced inclusion of a neuron-specific microexon, leading to diminished expression of the *Cpeb4* transcript that activates translation of mRNAs via polyadenylation under normal conditions	ASD	[99]
	Splicing Translation, mRNA stability, miRNA biogenesis	RBFOX1, RBFOX2 (RBM9), RBFOX3 (Neun)	RBFOX1 binds to the 3′-UTR of its target mRNAs and regulates: - Splicing of *Camk2d* and *Camk2g* mRNAs; - Stability of *Camk2a*, *Camk2b*, *Camk4*, and *Ppp3r1* mRNAs; - translational regulation by RBFOX2 and RBFOX3 (repression) - miRNA biogenesis. Altered splicing of RBFOX family proteins impairs their control of gene expression	ASD	[23,100,101,102]
	Transport, Translation	FMRP	CGG repeat expansion beyond 200 (>200) at the 5′-UTR of *FMR1* affects protein expression, resulting in dysregulated spatio-temporal transport/translation of dendritic mRNAs	FXS	[64]
	APA	NUDT21	Elevated amount of NUDT21, a subunit of pre-mRNA cleavage factor Im, due to copy number variation causes abnormal usage of polyadenylation sites, resulting in the generation of an inefficiently translated long isoform of MeCP2 protein.	Neuropsychia tric disease	[44]
Neuro degenerative diseases	Splicing	PRPF8	Mutated Huntingtin (HTT) traps PRPF8 (a splicing factor) to cause *CREB1* mis-splicing	HD	[18]
	Translation	HTT	Mutant HTT stalls ribosomes	HD	[72]
	Splicing	MBNL family proteins	RNA corresponding to expanded microsatellite repeats in *DMPK* traps MBNL-family proteins, impairing their normal function in splicing	DM	[103]
	Translation	ATAXIN-2	CAG expansion in the reading frame of *ATAXIN-2* causes loss of protein function that, under normal conditions, acts as an mRNA translation activator via polyadenylation	SCA2, ALS	[75]
	RAN Translation, Abnormal RNA structure (RNA foci)	Matrin-3	GGGGCC repeat expansion mutation in the *C9orf72* gene causes sequestration of Matrin-3 at the RNA foci and RAN translated peptides and loss of function of Matrin-3	FTLD, ALS	[104]
	mRNA stability, Splicing, Translation	nELAVL	nELAVL regulates disease-specific splicing of the pre-mRNAs *Picalm* and *Bin1* by incorporating exons 13 and 6a, respectively. The proteins corresponding to these spliced isoforms have been implicated in trafficking of amyloid precursor protein	AD	[105]
	Transport, Translation, miRNA biogenesis	TDP-43	- TDP-43-mediated axonal transport/translation of mRNAs such as *Nefl* and *Map1b* is adversely affected in diseased neurons expressing disease-specific mutant TDP-43; - TDP-43 has been implicated in FMRP co-regulation of mRNA transport/translation; - Nuclear localization of TDP-43 is affected in diseased neurons, altering its RNA-binding ability and the fate of target RNAs; - Normal TDP-43 function in cleaving certain pre-miRNAs via Drosha binding in the nucleus is impaired.	FTLD, ALS	[63,106,107,108]
	Transport, Translation	FUS	Normal FUS functions such as axonal transport/translation of mRNAs are adversely impacted in diseased neurons. Under disease conditions, the altered intracellular localization of FUS disrupts its functions as an RBP	FTLD, ALS	[109]
	Splicing, miRNA biogenesis	hnRNPs, MBNL1	mRNA corresponding to shorter CGG repeat expansions (<200) in the 5′UTR of *FMR1* sequester many RBPs, e.g., hnRNPs and MBNL1	Fragile X-associated tremor/ataxia syndrome (FXTAS)	[110]
	APA	α-synuclein	Presence of an extended 3′-UTR region in α-synuclein transcript impacts accumulation of α-synuclein protein that is redirected away from synaptic terminals towards mitochondria	PD	[48]

UTR—untranslated region; hnRNPs—heterogenous nuclear ribonuleoproteins.
